# SDG 3 measurement in integrated health system reform: a growing gap as 2030 approaches

**DOI:** 10.3389/frhs.2026.1845464

**Published:** 2026-04-23

**Authors:** Nurah Alamro

**Affiliations:** 1 Department of Family and Community Medicine, College of Medicine, King Saud University, Riyadh, Saudi Arabia; 2 Department of Family and Community Medicine, King Saud University Medical City, King Saud University, Riyadh, Saudi Arabia

**Keywords:** health policy, health system performance, health system reform, integrated health systems, policy evaluation, sustainable development goals, system-level evaluation

## Abstract

Integrated health system reforms are implemented across financing, service delivery, digital health, and governance, while SDG 3 monitoring relies on indicator-based frameworks designed for discrete interventions. This creates a misalignment between how reforms are implemented and how progress is assessed, particularly where outcomes emerge from interactions across system components. Using Saudi Arabia's Vision 2030 reform as an illustrative case, this analysis identifies three constraints: indicator-based measurement, limited evaluability of concurrent reforms, and data and governance limitations. These constraints limit the ability of policymakers to assess system performance, determine reform effectiveness, and allocate resources. Addressing this gap requires a shift toward system-level evaluation that integrates indicators across domains, strengthens data linkage, and aligns national and global monitoring frameworks.

## Introduction

Health system reform is increasingly implemented through coordinated changes across multiple domains, including governance, financing, service delivery, digital health, and prevention, creating challenges for monitoring and evaluating progress. In these contexts, reforms are not introduced as isolated interventions but as interconnected components of broader system transformation. While this approach reflects the complexity of contemporary health challenges, it complicates the attribution of observed changes in outcomes to specific policy actions (1–3).

This challenge is particularly relevant to the measurement of progress toward Sustainable Development Goal (SDG) 3. Current SDG monitoring frameworks are largely structured around discrete indicators linked to specific interventions, such as service coverage, utilisation, and selected health outcomes. These approaches assume that policy effects can be identified and measured independently. However, as health system reforms increasingly occur through interacting changes across domains, this assumption does not hold in integrated reform settings. While previous critiques have focused on fragmentation and limitations of individual indicators, this analysis highlights a distinct limitation: the inability of current measurement frameworks to capture interactions across concurrently implemented system-level reforms. This limitation is not addressed in current SDG 3 monitoring frameworks, which remain oriented toward assessing individual interventions rather than system-level interactions.

Saudi Arabia's health system reform under Vision 2030 provides a contemporary example of large-scale, integrated transformation. The reform is embedded within a programmatic architecture that emphasises performance monitoring and measurable targets. The National Transformation Program, introduced in 2016, established defined objectives and key performance indicators for the health sector, signalling a shift toward performance-based stewardship (4). This was followed by the Health Sector Transformation Program, which tracks system-level progress toward 2030 targets through structured monitoring frameworks (5).

Implementation has involved reforms across digital health, financing, and service delivery domains. Evidence documents the expansion of digital platforms, restructuring of service delivery models, and changes in financing and insurance coverage (6–9). These developments have been accompanied by expanded national performance measurement systems. The Ada'a programme, a national performance monitoring system, expanded from a limited set of hospitals and indicators in its early phase to a system covering public and private hospitals and primary healthcare centres across multiple performance domains by 2025, reflecting the institutionalisation of measurement and improvement mechanisms at scale (10).

At the same time, the evidence base remains more developed in documenting implementation processes and intermediate outputs than in assessing system-wide outcomes. Existing studies are predominantly qualitative and cross-sectional, enabling analysis of reform design and implementation but limiting causal inference and longitudinal assessment of impact (1–3). In addition, gaps in evaluation design, including limited availability of defined metrics in some areas of reform, constrain assessment of effects on health outcomes, equity, and financial protection.

This creates a divergence between implementation capacity and the ability to evaluate system-wide outcomes. While health systems are increasingly able to implement large-scale, system-wide reform, measurement approaches remain oriented toward discrete indicators and individual interventions. As a result, dimensions of system performance that arise from interactions across domains are not fully captured.

The Saudi experience illustrates both the feasibility of large-scale system transformation and the limitations of current measurement approaches in capturing such transformation. This highlights a structural challenge for the post-2030 agenda, requiring adaptation of how SDG 3 progress is defined, measured, and interpreted in complex reform settings. This analysis examines this challenge in the context of integrated health system reform and its implications for SDG 3 measurement.

## Policy options and implications

### SDG 3 measurement is not aligned with how reform is implemented

Current approaches to monitoring progress toward SDG 3 are structured around discrete indicators linked to individual interventions, including service coverage, utilisation, and selected health outcomes. This structure assumes that policy inputs can be separated and their effects measured independently. While appropriate for programme-based interventions, this approach does not reflect how contemporary health system reform is implemented. The evidence shows that reform is increasingly delivered through coordinated changes across financing, service delivery, digital platforms, and governance structures (6–9), illustrating how reforms are implemented concurrently rather than as isolated interventions. These changes operate together rather than independently, and outcomes arise from their interaction. This creates a mismatch between how progress is measured and how outcomes are produced. In practical terms, this constrains governments' ability to determine whether reforms are improving access, quality, and equity, which are central to SDG 3.

### Constraint 1: indicator-based measurement obscures system performance

SDG 3 measurement frameworks track performance through individual indicators. While these provide information on specific dimensions of the system, they do not capture how system components interact. In integrated reform settings, changes in outcomes are shaped by the combined effects of financing, digital access, and service delivery redesign. Evidence indicates concurrent expansion across these domains, including increases in digital service use and insurance coverage, as well as changes in care delivery models (6–9). In practice, these changes are implemented within the same system and time period, making it difficult to distinguish the contribution of individual interventions to observed outcomes. For example, simultaneous expansion of digital service platforms and insurance coverage can influence service utilisation patterns in ways that cannot be attributed to a single policy intervention. However, indicator-based measurement captures these changes separately, without reflecting how they influence one another. This constrains interpretation of progress. Policymakers can observe improvements in coverage or utilisation but cannot determine whether these reflect improved system performance, substitution effects, or temporary shifts in access. This constrains the ability to assess reform effectiveness, prioritise interventions, and allocate resources efficiently. It also reduces the ability of decision-makers to determine which components of reform should be expanded, modified, or discontinued.

### Constraint 2: rapid implementation exceeds evaluation capacity

Health system reforms are being implemented at a scale and pace that exceed the capacity of existing evaluation approaches. Evidence points to rapid expansion in service delivery models, digital platforms, and performance monitoring systems within reform programmes (6–9). However, the available evidence remains predominantly qualitative and cross-sectional, limiting the ability to assess causal relationships in settings where multiple interventions are introduced simultaneously (1–3). In some areas, evaluation metrics are not fully defined, further constraining assessment of impact. This creates a gap between implementation and evaluation. In applied settings, this is reflected in the absence of evaluation designs that can account for overlapping interventions introduced within short timeframes. Governments are able to deliver reform at scale but are less able to determine which components are effective, which populations benefit, and how resources should be allocated. This limits adaptive policy-making and reduces the capacity to optimise reform over time. It also constrains governments' ability to allocate resources based on evidence of effectiveness and to adjust reform strategies in real time.

### Constraint 3: data and governance systems limit system-level analysis

Effective evaluation of integrated reform requires data that are linked across financing, service delivery, and population health. However, evidence highlights fragmentation in data systems, limited interoperability, and challenges in coordinating information across institutional actors (7, 9). At the same time, governance arrangements often involve multiple public and private actors, increasing coordination requirements for data sharing, performance monitoring, and accountability. Variation in implementation across organisational units can further affect the consistency and comparability of data. As a result, system-level analysis is constrained. Policymakers are not able to assess how reforms affect equity, financial protection, or long-term outcomes across the population. This limits the ability to identify disparities, monitor unintended consequences, and ensure that reforms deliver intended benefits. It also limits accountability across institutions and reduces the capacity to monitor whether reforms are delivering equitable outcomes across populations.

### A framework for measuring integrated health system reform under SDG 3

The constraints identified above reflect a structural limitation in how progress is currently assessed. Measurement frameworks continue to rely on discrete indicators and are not sufficient to capture system-level reform dynamics, while reform is implemented as an integrated system. Addressing this gap requires a shift from indicator-based tracking to system-level evaluation (Table 1).

**Table 1:** Proposed measurement framework.

Dimension	What current SDG indicators capture	What is not captured in integrated reform	Policy implication
Coverage and utilisation	Service access and use	Interaction between financing, digital access, and care pathways	Integrate indicators to reflect combined effects of access, financing, and delivery
Financing	Spending levels and insurance coverage	Effects of purchasing models, incentives, and provider behaviour	Incorporate indicators on purchasing, incentives, and financial flows
Service delivery	Facility-based outputs	Coordination across levels of care and continuity of care	Measure integration across primary, secondary, and tertiary care
Digital health	Platform access and use	Interoperability and influence on care pathways	Include indicators on data integration and continuity of digital-enabled care
Governance and performance	Selected KPIs	Coordination across actors and implementation fidelity	Assess governance effectiveness and system coordination mechanisms
Equity	Aggregate population indicators	Distributional effects across regions and population groups	Systematically disaggregate data and monitor equity outcomes

This framework shifts the focus from measuring individual components to assessing how system elements interact to produce outcomes. It can be operationalised through integrated performance dashboards that link financing, service delivery, and population health indicators within unified monitoring systems. Such approaches are increasingly reflected in national health system performance platforms and evolving global discussions on integrated monitoring of SDG progress. It also highlights the need for linked data systems, longitudinal analysis, and equity-sensitive measurement. Operationalising such a framework would require alignment with existing global and national monitoring systems. At the global level, this includes integration with WHO-led SDG indicator frameworks and adaptation of reporting mechanisms coordinated through the United Nations system. At the national level, it requires incorporation into health system performance dashboards and data platforms that link financing, service delivery, and population health. This would enable countries to move beyond reporting individual indicators toward assessing system performance in a way that supports policy decisions, resource allocation, and accountability. Governments should prioritise integration of SDG indicators into national performance monitoring systems, strengthen interoperability across data platforms, and incorporate cross-domain metrics that reflect interactions between financing, service delivery, and population health.

### Actionable recommendations

- Integrate SDG indicators across financing, service delivery, and population health domains to reflect system-level interactions.
- Strengthen data linkage and interoperability across health system components to enable system-level analysis.
- Incorporate cross-domain metrics that capture interactions between service delivery, financing, and health outcomes.
- Align national performance monitoring systems with global SDG reporting frameworks to support integrated evaluation.
- Use system-level performance dashboards to support policy decision-making, resource allocation, and accountability.

## Conclusions

The evidence shows that current SDG 3 measurement approaches are not aligned with the realities of integrated health system reform. This represents a structural limitation in the current SDG 3 monitoring approach. This is particularly relevant as global discussions increasingly focus on the post-2030 development agenda, with growing recognition within the United Nations system of the need to adapt monitoring and implementation approaches for the next phase of sustainable development (11). As a result, progress can be observed but not fully interpreted. This limits the ability of policymakers to assess system performance, prioritise interventions, and allocate resources effectively. At a broader level, this misalignment also affects the capacity of SDG 3 to function as a global framework for monitoring progress, coordinating action, and ensuring accountability across countries. Without such adaptation, SDG 3 monitoring is likely to misrepresent system performance and reduce its effectiveness as a tool for guiding policy, allocating resources, and ensuring accountability in increasingly complex health system reform settings.
